# Supplementary material for: The Peroxisome Proliferator-Activated Receptor α- Agonist Gemfibrozil Promotes Defense Against Mycobacterium abscessus Infections
Source: Cells. 2020 Mar 6;9(3):648. doi: 10.3390/cells9030648 (PMC7140404; doi:10.3390/cells9030648)
Supplement: Supplementary file 1 [file cells-09-00648-s001.zip › Revised supplementary Figure legends ver1.1.docx]

**Figure S1.** **PPARα suppresses activated neutrophil infiltration in Mabc-infected lung tissue.**

*Ppara*^+/+^ (n = 7) and *Ppara*^-/-^ (n = 5) mice were infected iv with Mabc (1 × 10^7^ CFU), and lung tissues were stained with MPO (red) and DAPI (nuclei; blue). Scale bars, 50 μm. **Average mean fluorescence intensities (MFIs) of** MPO. Data are means ± SD of three independent experiments. Images are representative of three independent experiments. *** p *<* 0.001. UI, uninfected.

**Figure S2.** **In vitro activity of gemfibrozil.**

The activity of gemfibrozil (GEM) against Mabc CIP 104536T in comparison with clarithromycin (CLR) in cation-adjusted Mueller–Hinton (CAMH) and 7H9 broth.

**Figure S3.** **Treatment of macrophages with GEM, but not GW7647, enhances nuclear translocation of TFEB during Mabc infection.**

BMDMs were infected with Mabc (MOI of 5) for 3 h and treated with gemfibrozil (GEM; 100 μM) or GW7647 (20 μM). TFEB nuclear translocation was assessed by confocal microscopy and calculated nuclear translocation of TFEB. Data are means ± SD of three independent experiments. *p < 0.05. ns, not significant; U, untreated.
