# Supplementary material for: The Peroxisome Proliferator-Activated Receptor α- Agonist Gemfibrozil Promotes Defense Against Mycobacterium abscessus Infections
Source: Cells. 2020 Mar 6;9(3):648. doi: 10.3390/cells9030648 (PMC7140404; doi:10.3390/cells9030648)
Supplement: Supplementary file 1 [file cells-09-00648-s001.zip › Supplementary table 1.docx]

| Genes | Primer | Sequences |
| --- | --- | --- |
| *Tnf* | Forward  Reverse | 5′-CCCACGTCGTAGCAAACCAC-3′  5′-GCAGCCTTGTCCCTTGAAGA-3′ |
| *Il6* | Forward  Reverse | 5′-ACAAAGCCAGAGTCCTTCAGA-3′  5′-TGGTCCTTAGCCACTCCTTC-3′ |
| *Il12p40* | Forward  Reverse | 5′-AGGTCACACTGGACCAAAGG-3′  5′-TGGTTTGATGATGTCCCTGA-3′ |
| *Il1b* | Forward  Reverse | 5′-TGACGGACCCCAAAAGATGA-3′  5′-AAAGACACAGGTAGCTGCCA-3′ |
| *Il10* | Forward  Reverse | 5′-GCTCTTGCACTACCAAAGCC-3′  5′-CTGCTGATCCTCATGCCAGT-3′ |
| *Ccl5* | Forward  Reverse | 5′-CCTCACCATCCTCACTG-3′  5′-TCTTCTCTGGGTTGGCACAC-3′ |
| *Ccl2* | Forward  Reverse | 5′-ACTCAAGCCAGCTCTCTCTT-3′  5′-TTCCTTCTTGGGGTCAGCAC-3′ |
| *Cxcl10* | Forward  Reverse | 5′-AAGTGCTGCCGTCATTTTCT-3′  5′-CATTCTTTTTCATCGTGGCA-3′ |
| *Ccl12* | Forward  Reverse | 5′-CTTCTATGCCTCCTGCTCAT-3′  5′-CGGACGTGAATCTTCTGCTT-3′ |
| *Cxcl5* | Forward  Reverse | 5′-GCACTCGCAGTGGAAAGAAC-3′  5′-CGTGGGTGGAGAGAATCAGC-3′ |
| *Gapdh* | Forward  Reverse | 5′-TGGCAAAGTGGAGATTGTTGCC-3′  5′-AAGATGGTGATGGGCTTCCCG-3′ |

**Supplementary Table 1.** Primers used in this study.
