# Supplementary figures and images for: The Peroxisome Proliferator-Activated Receptor α- Agonist Gemfibrozil Promotes Defense Against Mycobacterium abscessus Infections
Source: Cells. 2020 Mar 6;9(3):648. doi: 10.3390/cells9030648 (PMC7140404; doi:10.3390/cells9030648)

Figure S1

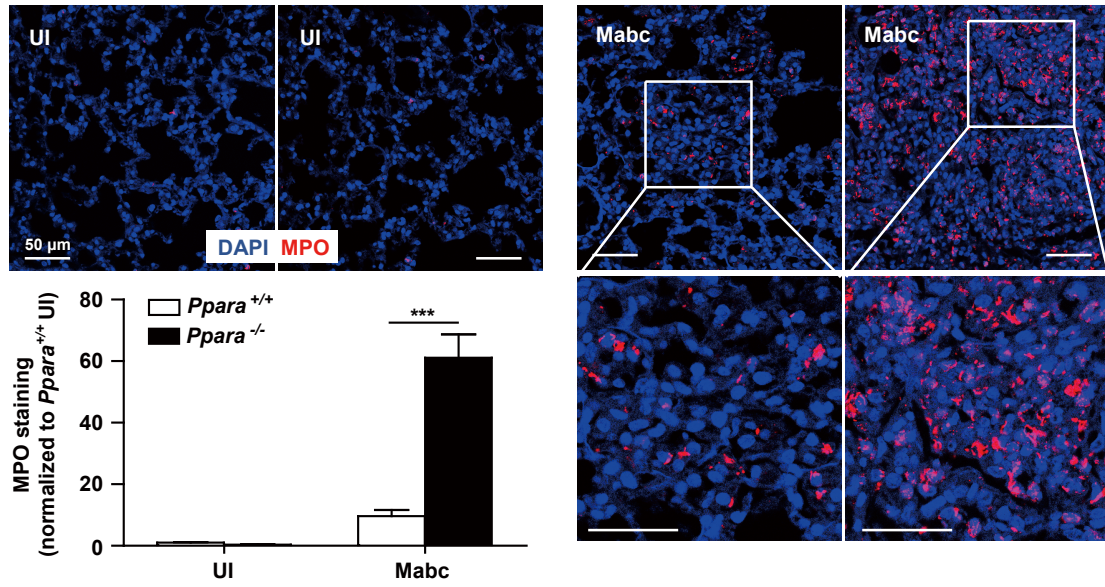

Figure S2

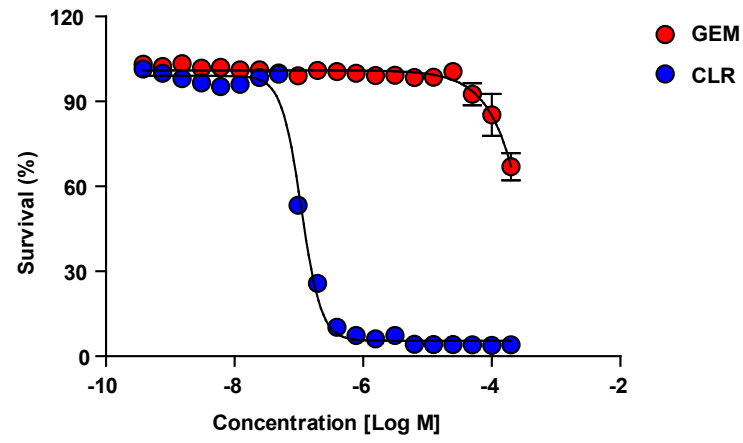

**Figure S3**

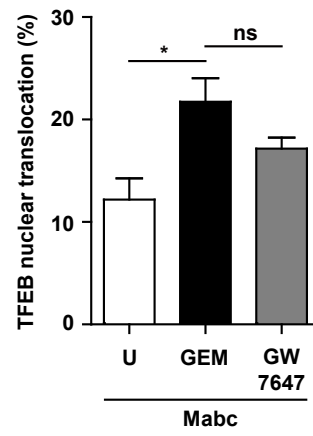

Supplement: Supplementary file 1 [file cells-09-00648-s001.zip › Supple Figures.pdf]
